# Methyl 1-ethyl-3′-[hy­droxy(naphthalen-1-yl)meth­yl]-1′-methyl-2-oxo­spiro­[indo­line-3,2′-pyrrolidine]-3′-carboxyl­ate

**DOI:** 10.1107/S1600536814007065

**Published:** 2014-04-12

**Authors:** Vinodhkumar Vijayakumar, Gunther H. Peters, M. Suresh, Raghunathan Raghavachary, G. Jagadeesan

**Affiliations:** a Department of Life Sciences and Chemistry, Roskilde University, DK-4000 Roskilde, Denmark; b Department of Chemistry, Technical University of Denmark, 2800 Kgs. Lyngby, Denmark; c Department of Organic Chemistry, University of Madras, Guindy Campus, Chennai 600 025, India; d Department of Physics, Presidency College, Chennai 600 005, India

## Abstract

In the title compound, C_27_H_28_N_2_O_4_, the pyrrolidine ring adopts a twist conformation. The plane of the indole ring is almost perpendicular to that of the pyrrolidine ring, making a dihedral angle of 88.50 (6)°. The planes of the naphthyl ring system and the pyrrolidine ring are tilted by an angle of 55.86 (5)°. The mol­ecular conformation is stabilized by intra­molecular O—H⋯O and O—H⋯N hydrogen bonds.

## Related literature   

For general background to spiro compounds and their biological activity, see: Pradhan *et al.* (2006 ▶); For uses of pyrrolidine derivative, see: Amal Raj *et al.* (2003 ▶); For conformation studies, see: Nardelli (1983 ▶).
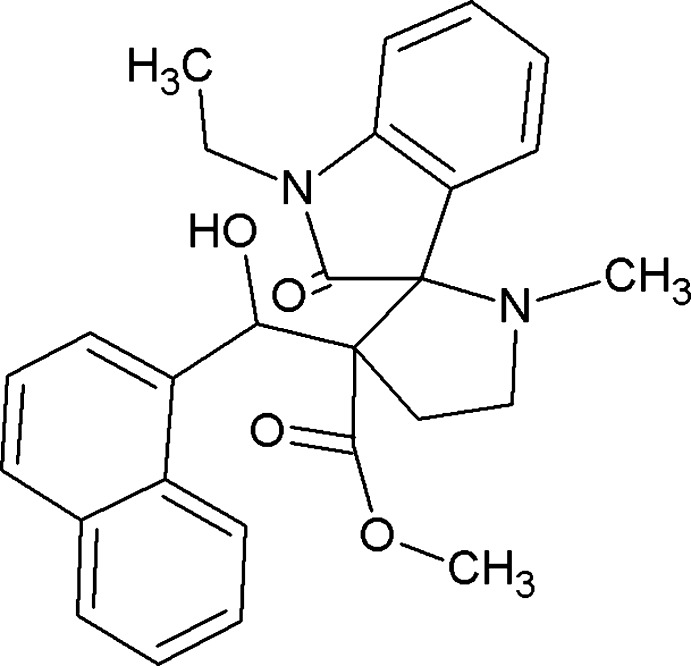


## Experimental   

### 

#### Crystal data  

C_27_H_28_N_2_O_4_
*M* *_r_* = 444.51
Orthorhombic, 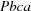

*a* = 16.7802 (3) Å
*b* = 14.6690 (3) Å
*c* = 18.4735 (4) Å
*V* = 4547.23 (16) Å^3^
*Z* = 8
Mo *K*α radiation
μ = 0.09 mm^−1^
*T* = 293 K
0.25 × 0.20 × 0.20 mm

#### Data collection  

Bruker Kappa APEXII CCD diffractometer
Absorption correction: multi-scan (*SADABS*; Bruker, 2004 ▶) *T*_min_ = 0.979, *T*_max_ = 0.983
44640 measured reflections
4636 independent reflections
3429 reflections with *I* > 2σ(*I*)
*R*_int_ = 0.041

#### Refinement  

*R*[*F* ^2^ > 2σ(*F* ^2^)] = 0.039
*wR*(*F* ^2^) = 0.111
*S* = 1.02
4636 reflections
299 parameters
H-atom parameters constrained
Δρ_max_ = 0.22 e Å^−3^
Δρ_min_ = −0.14 e Å^−3^

### 

Data collection: *APEX2* (Bruker, 2004 ▶); cell refinement: *APEX2* and *SAINT* (Bruker, 2004 ▶); data reduction: *SAINT* and *XPREP* (Bruker, 2004 ▶); program(s) used to solve structure: *SHELXS97* (Sheldrick, 2008 ▶); program(s) used to refine structure: *SHELXL97* (Sheldrick, 2008 ▶); molecular graphics: *ORTEP-3 for Windows* (Farrugia, 2012 ▶); software used to prepare material for publication: *PLATON* (Spek, 2009 ▶).

## Supplementary Material

Crystal structure: contains datablock(s) I, 2R. DOI: 10.1107/S1600536814007065/bt6950sup1.cif

Structure factors: contains datablock(s) I. DOI: 10.1107/S1600536814007065/bt6950Isup2.hkl

Supporting information file. DOI: 10.1107/S1600536814007065/bt6950Isup3.cml

CCDC reference: 993217

Additional supporting information:  crystallographic information; 3D view; checkCIF report

## Figures and Tables

**Table 1:** Hydrogen-bond geometry (Å, °)

*D*—H⋯*A*	*D*—H	H⋯*A*	*D*⋯*A*	*D*—H⋯*A*
O1—H1⋯O4	0.82	2.37	2.9121 (16)	124
O1—H1⋯N1	0.82	2.39	2.9439 (17)	126

**Fig. 1.**
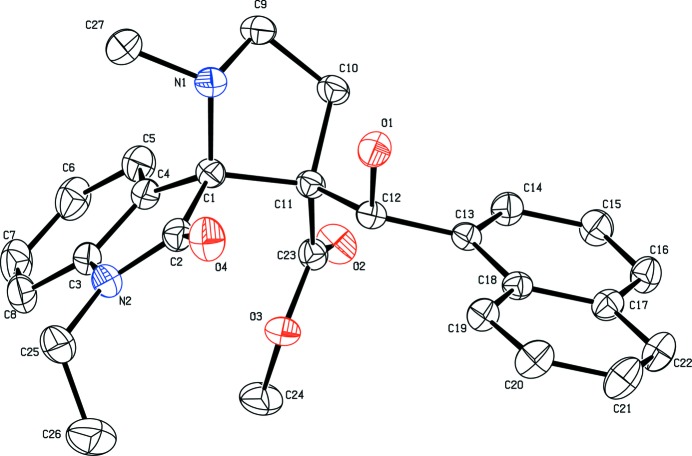
The molecular structure of the title compound, Displacement ellipsoids are drawn at the 30% probability level, H atoms have been omitted for clarity.

**Fig. 2.**
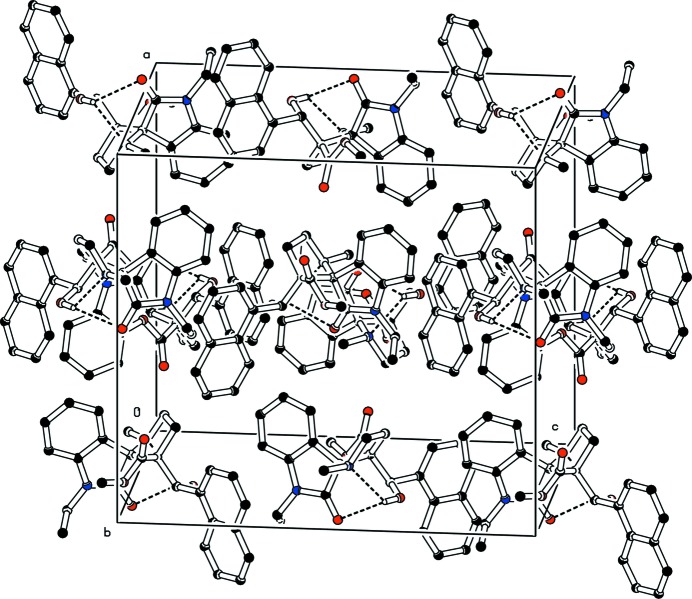
Crystal packing of the title compound, Hydrogen bonds are shown as dashed lines. For the sake of clarity, H atoms not involved in the interactions have been omitted.

## supplementary crystallographic information

### 1. Comment

Spiro compounds have received considerable interest due to their biological properties (Pradhan *et al.*, 2006). In addition, pyrrolidine derivatives are found to have anticonvulsant, antimicrobial and antifungal activities against various pathogens (Amal Raj *et al.*, 2003). In view of their importance, the crystal structure determination of the title compound was carried out and the results are presented herein. In the title molecule (Fig. 1) the five-membered pyrrolidine ring [DS (N1) = 0.101 (1) Å and D2 (C10) = 0.051 (9) Å] adopts a twist conformation defined by the above asymmetry parameters (Nardelli, 1983). The indole ring (C1—C8/N2) is almost perpendicular to the pyrrolidine ring with dihedral angle of 88.50 (6)°. The naphthyl and pyrrolidine rings are tilted by an angle of 55.86 (5)°. The molecular conformation is stabilized by an intramolecular O—H···O and O—H···N hydrogen bond (Fig. 2 and Table 1).

### 2. Experimental

A mixture of methyl 2-(hydroxy(naphthalen-1-yl)methyl)acrylate (1 mmol), *N*-ethyl isatin (1.1 mmol) and sarcosine (1.1 mmol) was refluxed in methanol until completion of the reaction was evidenced by TLC analysis. After completion of the reaction the solvent was evaporated under reduced pressure. The reaction mixture was dissolved in ethyl acetate and washed with water followed by brine solution. The organic layer was separated and evaporated under reduced pressure. The crude mixture was purified by column chromatography using ethyl acetate and hexane as eluent (3: 7). The product was dissolved in ethyl acetate and heated for two minutes. The resulting solution was subjected to crystallization by slow evaporation of the solvent for 48 h resulting in the formation of single crystals

### 3. Refinement

All H atoms were positioned geometrically, with C–H = 0.93–0.97 Å and constrained to ride on their parent atom with *U*_iso_(H) = 1.5*U*_eq_(O,C) for methyl H atoms and 1.2*U*_eq_(C) for other H atoms.

### Crystal data

**Table:**

C_27_H_28_N_2_O_4_	*F*(000) = 1888
*M**_r_* = 444.51	*D*_x_ = 1.299 Mg m^−^^3^
Orthorhombic, *P**b**c**a*	Mo *K*α radiation, λ = 0.71073 Å
Hall symbol: -P 2ac 2ab	Cell parameters from 8834 reflections
*a* = 16.7802 (3) Å	θ = 2.1–31.2°
*b* = 14.6690 (3) Å	µ = 0.09 mm^−^^1^
*c* = 18.4735 (4) Å	*T* = 293 K
*V* = 4547.23 (16) Å^3^	Block, colourless
*Z* = 8	0.25 × 0.20 × 0.20 mm

### Data collection

**Table:**

Bruker Kappa APEXII CCD diffractometer	4636 independent reflections
Radiation source: fine-focus sealed tube	3429 reflections with *I* > 2σ(*I*)
Graphite monochromator	*R*_int_ = 0.041
ω and φ scan	θ_max_ = 26.4°, θ_min_ = 2.2°
Absorption correction: multi-scan (*SADABS*; Bruker, 2004)	*h* = −20→18
*T*_min_ = 0.979, *T*_max_ = 0.983	*k* = −18→18
44640 measured reflections	*l* = −23→23

### Refinement

**Table:**

Refinement on *F*^2^	Secondary atom site location: difference Fourier map
Least-squares matrix: full	Hydrogen site location: inferred from neighbouring sites
*R*[*F*^2^ > 2σ(*F*^2^)] = 0.039	H-atom parameters constrained
*wR*(*F*^2^) = 0.111	*w* = 1/[σ^2^(*F*_o_^2^) + (0.0509*P*)^2^ + 1.1973*P*] where *P* = (*F*_o_^2^ + 2*F*_c_^2^)/3
*S* = 1.02	(Δ/σ)_max_ = 0.001
4636 reflections	Δρ_max_ = 0.22 e Å^−^^3^
299 parameters	Δρ_min_ = −0.14 e Å^−^^3^
0 restraints	Extinction correction: *SHELXL97* (Sheldrick, 2008), Fc^*^=kFc[1+0.001xFc^2^λ^3^/sin(2θ)]^-1/4^
Primary atom site location: structure-invariant direct methods	Extinction coefficient: 0.0061 (4)

### Special details

**Table:**

Geometry. All e.s.d.'s (except the e.s.d. in the dihedral angle between two l.s. planes) are estimated using the full covariance matrix. The cell e.s.d.'s are taken into account individually in the estimation of e.s.d.'s in distances, angles and torsion angles; correlations between e.s.d.'s in cell parameters are only used when they are defined by crystal symmetry. An approximate (isotropic) treatment of cell e.s.d.'s is used for estimating e.s.d.'s involving l.s. planes.
Refinement. Refinement of *F*^2^ against ALL reflections. The weighted *R*-factor *wR* and goodness of fit *S* are based on *F*^2^, conventional *R*-factors *R* are based on *F*, with *F* set to zero for negative *F*^2^. The threshold expression of *F*^2^ > σ(*F*^2^) is used only for calculating *R*-factors(gt) *etc*. and is not relevant to the choice of reflections for refinement. *R*-factors based on *F*^2^ are statistically about twice as large as those based on *F*, and *R*- factors based on ALL data will be even larger.

### Fractional atomic coordinates and isotropic or equivalent isotropic displacement parameters (Å^2^)

**Table:**

	*x*	*y*	*z*	*U*_iso_*/*U*_eq_	
O1	0.96460 (6)	0.22318 (7)	0.34518 (6)	0.0455 (3)	
H1	0.9670	0.1849	0.3775	0.068*	
O3	0.90834 (6)	0.42866 (7)	0.49176 (5)	0.0417 (3)	
O2	0.78729 (7)	0.43761 (8)	0.44130 (6)	0.0539 (3)	
N1	0.86301 (8)	0.14862 (9)	0.46083 (7)	0.0430 (3)	
O4	1.02618 (7)	0.21025 (9)	0.49220 (6)	0.0573 (3)	
N2	0.96210 (8)	0.25963 (9)	0.59423 (7)	0.0456 (3)	
C18	1.01590 (8)	0.40195 (9)	0.28012 (7)	0.0332 (3)	
C13	0.94194 (8)	0.37632 (10)	0.31325 (7)	0.0328 (3)	
C12	0.94494 (8)	0.30940 (9)	0.37557 (7)	0.0332 (3)	
H12	0.9891	0.3276	0.4071	0.040*	
C17	1.01480 (9)	0.46284 (10)	0.22021 (8)	0.0388 (3)	
C3	0.88393 (10)	0.28059 (10)	0.61479 (8)	0.0432 (4)	
C1	0.88143 (9)	0.23837 (10)	0.49136 (7)	0.0360 (3)	
C4	0.83254 (10)	0.26839 (10)	0.55663 (8)	0.0397 (4)	
C10	0.79905 (9)	0.25417 (11)	0.38403 (8)	0.0410 (4)	
H10A	0.7503	0.2892	0.3892	0.049*	
H10B	0.8108	0.2476	0.3329	0.049*	
C19	1.09071 (9)	0.36786 (11)	0.30294 (8)	0.0395 (4)	
H19	1.0931	0.3281	0.3421	0.047*	
C11	0.86884 (8)	0.30242 (10)	0.42346 (7)	0.0333 (3)	
C23	0.84805 (9)	0.39681 (10)	0.45129 (7)	0.0364 (3)	
C16	0.94110 (10)	0.49640 (11)	0.19414 (9)	0.0459 (4)	
H16	0.9401	0.5363	0.1550	0.055*	
C14	0.87276 (9)	0.41091 (11)	0.28530 (8)	0.0415 (4)	
H14	0.8245	0.3943	0.3063	0.050*	
C20	1.15904 (9)	0.39227 (12)	0.26867 (10)	0.0503 (4)	
H20	1.2075	0.3688	0.2844	0.060*	
C2	0.96610 (9)	0.23489 (11)	0.52364 (8)	0.0417 (4)	
C9	0.79001 (10)	0.16118 (11)	0.41925 (9)	0.0471 (4)	
H9A	0.7437	0.1598	0.4507	0.057*	
H9B	0.7845	0.1139	0.3829	0.057*	
C8	0.85759 (13)	0.31094 (12)	0.68131 (9)	0.0583 (5)	
H8	0.8926	0.3189	0.7198	0.070*	
C21	1.15740 (10)	0.45237 (12)	0.20990 (10)	0.0569 (5)	
H21	1.2046	0.4686	0.1869	0.068*	
C5	0.75264 (10)	0.28726 (12)	0.56506 (9)	0.0490 (4)	
H5	0.7175	0.2798	0.5266	0.059*	
C22	1.08714 (10)	0.48694 (12)	0.18645 (9)	0.0500 (4)	
H22	1.0866	0.5272	0.1475	0.060*	
C27	0.85972 (13)	0.07413 (12)	0.51293 (10)	0.0628 (5)	
H27A	0.9099	0.0696	0.5376	0.094*	
H27B	0.8489	0.0180	0.4880	0.094*	
H27C	0.8182	0.0857	0.5474	0.094*	
C24	0.89186 (12)	0.50715 (12)	0.53604 (10)	0.0605 (5)	
H24A	0.9391	0.5241	0.5620	0.091*	
H24B	0.8502	0.4928	0.5698	0.091*	
H24C	0.8753	0.5569	0.5058	0.091*	
C6	0.72542 (12)	0.31771 (13)	0.63193 (10)	0.0595 (5)	
H6	0.6716	0.3305	0.6383	0.071*	
C25	1.03135 (11)	0.26205 (13)	0.64167 (10)	0.0607 (5)	
H25A	1.0698	0.2172	0.6252	0.073*	
H25B	1.0150	0.2452	0.6902	0.073*	
C15	0.87216 (10)	0.47049 (11)	0.22601 (9)	0.0471 (4)	
H15	0.8239	0.4924	0.2084	0.057*	
C7	0.77716 (14)	0.32907 (13)	0.68843 (10)	0.0647 (5)	
H7	0.7577	0.3495	0.7327	0.078*	
C26	1.07060 (14)	0.35314 (17)	0.64422 (14)	0.0871 (7)	
H26A	1.1159	0.3505	0.6759	0.131*	
H26B	1.0334	0.3976	0.6619	0.131*	
H26C	1.0877	0.3699	0.5965	0.131*	

### Atomic displacement parameters (Å^2^)

**Table:**

	*U*^11^	*U*^22^	*U*^33^	*U*^12^	*U*^13^	*U*^23^
O1	0.0565 (7)	0.0361 (6)	0.0438 (6)	0.0055 (5)	0.0104 (5)	−0.0025 (5)
O3	0.0426 (6)	0.0372 (6)	0.0452 (6)	−0.0019 (4)	−0.0030 (5)	−0.0087 (5)
O2	0.0459 (7)	0.0586 (7)	0.0571 (7)	0.0166 (5)	−0.0053 (5)	−0.0069 (6)
N1	0.0528 (8)	0.0357 (7)	0.0404 (7)	−0.0048 (6)	−0.0031 (6)	−0.0018 (6)
O4	0.0428 (7)	0.0746 (9)	0.0544 (7)	0.0114 (6)	−0.0006 (6)	0.0086 (6)
N2	0.0503 (8)	0.0489 (8)	0.0377 (7)	−0.0043 (6)	−0.0115 (6)	0.0051 (6)
C18	0.0329 (7)	0.0321 (7)	0.0347 (7)	−0.0002 (6)	0.0017 (6)	−0.0043 (6)
C13	0.0315 (8)	0.0346 (8)	0.0324 (7)	−0.0005 (6)	−0.0002 (6)	−0.0042 (6)
C12	0.0311 (7)	0.0341 (8)	0.0343 (7)	−0.0009 (6)	0.0005 (6)	−0.0036 (6)
C17	0.0396 (8)	0.0348 (8)	0.0421 (8)	0.0004 (6)	0.0047 (7)	−0.0007 (7)
C3	0.0564 (10)	0.0392 (9)	0.0339 (8)	−0.0044 (7)	−0.0014 (7)	0.0047 (6)
C1	0.0372 (8)	0.0376 (8)	0.0331 (7)	−0.0024 (6)	−0.0001 (6)	−0.0020 (6)
C4	0.0482 (9)	0.0374 (8)	0.0336 (7)	−0.0064 (7)	0.0031 (7)	0.0016 (6)
C10	0.0358 (8)	0.0504 (9)	0.0367 (8)	−0.0091 (7)	−0.0033 (6)	−0.0035 (7)
C19	0.0349 (8)	0.0406 (8)	0.0431 (8)	0.0018 (6)	0.0023 (6)	0.0022 (7)
C11	0.0305 (7)	0.0377 (8)	0.0316 (7)	−0.0027 (6)	−0.0013 (6)	−0.0024 (6)
C23	0.0349 (8)	0.0412 (8)	0.0330 (7)	0.0003 (6)	0.0017 (6)	0.0004 (6)
C16	0.0464 (10)	0.0457 (9)	0.0454 (9)	0.0022 (7)	−0.0011 (7)	0.0107 (7)
C14	0.0317 (8)	0.0492 (9)	0.0434 (8)	−0.0022 (6)	0.0006 (6)	0.0042 (7)
C20	0.0327 (8)	0.0506 (10)	0.0677 (11)	0.0055 (7)	0.0058 (8)	0.0065 (8)
C2	0.0435 (9)	0.0415 (9)	0.0402 (8)	−0.0013 (7)	−0.0030 (7)	0.0068 (7)
C9	0.0492 (10)	0.0460 (9)	0.0461 (9)	−0.0123 (7)	−0.0028 (7)	−0.0066 (7)
C8	0.0876 (15)	0.0544 (11)	0.0328 (8)	−0.0036 (10)	−0.0018 (9)	0.0025 (8)
C21	0.0402 (10)	0.0541 (11)	0.0765 (12)	0.0031 (8)	0.0196 (9)	0.0147 (9)
C5	0.0477 (10)	0.0547 (10)	0.0447 (9)	−0.0037 (7)	0.0072 (7)	0.0012 (8)
C22	0.0493 (10)	0.0453 (10)	0.0553 (10)	0.0024 (7)	0.0137 (8)	0.0113 (8)
C27	0.0863 (15)	0.0439 (10)	0.0583 (11)	−0.0106 (9)	−0.0059 (10)	0.0068 (8)
C24	0.0749 (13)	0.0447 (10)	0.0621 (11)	−0.0021 (9)	−0.0012 (10)	−0.0205 (9)
C6	0.0653 (12)	0.0578 (11)	0.0553 (11)	0.0042 (9)	0.0214 (9)	0.0052 (9)
C25	0.0649 (12)	0.0647 (12)	0.0525 (10)	−0.0033 (9)	−0.0249 (9)	0.0088 (9)
C15	0.0379 (9)	0.0528 (10)	0.0507 (9)	0.0029 (7)	−0.0083 (7)	0.0086 (8)
C7	0.0926 (16)	0.0620 (12)	0.0394 (9)	0.0073 (11)	0.0194 (10)	0.0017 (8)
C26	0.0750 (15)	0.0904 (17)	0.0959 (17)	−0.0253 (12)	−0.0234 (13)	−0.0054 (14)

### Geometric parameters (Å, º)

**Table:**

O1—C12	1.4224 (17)	C11—C23	1.518 (2)
O1—H1	0.8200	C16—C15	1.353 (2)
O3—C23	1.3418 (17)	C16—H16	0.9300
O3—C24	1.4392 (19)	C14—C15	1.401 (2)
O2—C23	1.1965 (17)	C14—H14	0.9300
N1—C27	1.457 (2)	C20—C21	1.399 (2)
N1—C9	1.458 (2)	C20—H20	0.9300
N1—C1	1.4652 (19)	C9—H9A	0.9700
O4—C2	1.2185 (19)	C9—H9B	0.9700
N2—C2	1.355 (2)	C8—C7	1.382 (3)
N2—C3	1.400 (2)	C8—H8	0.9300
N2—C25	1.456 (2)	C21—C22	1.355 (2)
C18—C19	1.415 (2)	C21—H21	0.9300
C18—C17	1.422 (2)	C5—C6	1.391 (2)
C18—C13	1.4339 (19)	C5—H5	0.9300
C13—C14	1.368 (2)	C22—H22	0.9300
C13—C12	1.514 (2)	C27—H27A	0.9600
C12—C11	1.5570 (19)	C27—H27B	0.9600
C12—H12	0.9800	C27—H27C	0.9600
C17—C22	1.410 (2)	C24—H24A	0.9600
C17—C16	1.416 (2)	C24—H24B	0.9600
C3—C8	1.380 (2)	C24—H24C	0.9600
C3—C4	1.389 (2)	C6—C7	1.368 (3)
C1—C4	1.523 (2)	C6—H6	0.9300
C1—C2	1.542 (2)	C25—C26	1.490 (3)
C1—C11	1.581 (2)	C25—H25A	0.9700
C4—C5	1.378 (2)	C25—H25B	0.9700
C10—C9	1.519 (2)	C15—H15	0.9300
C10—C11	1.5501 (19)	C7—H7	0.9300
C10—H10A	0.9700	C26—H26A	0.9600
C10—H10B	0.9700	C26—H26B	0.9600
C19—C20	1.358 (2)	C26—H26C	0.9600
C19—H19	0.9300		
			
C12—O1—H1	109.5	C15—C14—H14	118.9
C23—O3—C24	116.77 (12)	C19—C20—C21	120.76 (15)
C27—N1—C9	114.27 (13)	C19—C20—H20	119.6
C27—N1—C1	115.31 (12)	C21—C20—H20	119.6
C9—N1—C1	105.46 (12)	O4—C2—N2	125.39 (15)
C2—N2—C3	111.49 (13)	O4—C2—C1	126.01 (14)
C2—N2—C25	123.11 (15)	N2—C2—C1	108.53 (13)
C3—N2—C25	125.40 (14)	N1—C9—C10	104.79 (12)
C19—C18—C17	117.71 (13)	N1—C9—H9A	110.8
C19—C18—C13	123.22 (13)	C10—C9—H9A	110.8
C17—C18—C13	119.05 (13)	N1—C9—H9B	110.8
C14—C13—C18	118.43 (13)	C10—C9—H9B	110.8
C14—C13—C12	123.76 (13)	H9A—C9—H9B	108.9
C18—C13—C12	117.77 (12)	C3—C8—C7	117.37 (17)
O1—C12—C13	106.51 (11)	C3—C8—H8	121.3
O1—C12—C11	110.86 (11)	C7—C8—H8	121.3
C13—C12—C11	116.59 (11)	C22—C21—C20	120.08 (15)
O1—C12—H12	107.5	C22—C21—H21	120.0
C13—C12—H12	107.5	C20—C21—H21	120.0
C11—C12—H12	107.5	C4—C5—C6	118.98 (17)
C22—C17—C16	120.97 (14)	C4—C5—H5	120.5
C22—C17—C18	119.38 (14)	C6—C5—H5	120.5
C16—C17—C18	119.64 (13)	C21—C22—C17	120.94 (15)
C8—C3—C4	122.11 (17)	C21—C22—H22	119.5
C8—C3—N2	127.79 (16)	C17—C22—H22	119.5
C4—C3—N2	110.09 (13)	N1—C27—H27A	109.5
N1—C1—C4	116.80 (12)	N1—C27—H27B	109.5
N1—C1—C2	108.27 (12)	H27A—C27—H27B	109.5
C4—C1—C2	101.51 (12)	N1—C27—H27C	109.5
N1—C1—C11	101.56 (11)	H27A—C27—H27C	109.5
C4—C1—C11	112.59 (12)	H27B—C27—H27C	109.5
C2—C1—C11	116.72 (12)	O3—C24—H24A	109.5
C5—C4—C3	119.39 (14)	O3—C24—H24B	109.5
C5—C4—C1	132.19 (14)	H24A—C24—H24B	109.5
C3—C4—C1	108.37 (13)	O3—C24—H24C	109.5
C9—C10—C11	106.51 (12)	H24A—C24—H24C	109.5
C9—C10—H10A	110.4	H24B—C24—H24C	109.5
C11—C10—H10A	110.4	C7—C6—C5	120.56 (18)
C9—C10—H10B	110.4	C7—C6—H6	119.7
C11—C10—H10B	110.4	C5—C6—H6	119.7
H10A—C10—H10B	108.6	N2—C25—C26	113.18 (16)
C20—C19—C18	121.12 (14)	N2—C25—H25A	108.9
C20—C19—H19	119.4	C26—C25—H25A	108.9
C18—C19—H19	119.4	N2—C25—H25B	108.9
C23—C11—C10	113.72 (12)	C26—C25—H25B	108.9
C23—C11—C12	108.73 (11)	H25A—C25—H25B	107.8
C10—C11—C12	112.50 (11)	C16—C15—C14	120.62 (15)
C23—C11—C1	107.70 (11)	C16—C15—H15	119.7
C10—C11—C1	101.67 (11)	C14—C15—H15	119.7
C12—C11—C1	112.35 (11)	C6—C7—C8	121.59 (17)
O2—C23—O3	123.65 (14)	C6—C7—H7	119.2
O2—C23—C11	126.86 (14)	C8—C7—H7	119.2
O3—C23—C11	109.47 (12)	C25—C26—H26A	109.5
C15—C16—C17	120.09 (14)	C25—C26—H26B	109.5
C15—C16—H16	120.0	H26A—C26—H26B	109.5
C17—C16—H16	120.0	C25—C26—H26C	109.5
C13—C14—C15	122.18 (14)	H26A—C26—H26C	109.5
C13—C14—H14	118.9	H26B—C26—H26C	109.5
			
C19—C18—C13—C14	−177.76 (14)	C4—C1—C11—C10	−91.23 (14)
C17—C18—C13—C14	0.7 (2)	C2—C1—C11—C10	151.93 (12)
C19—C18—C13—C12	−0.1 (2)	N1—C1—C11—C12	−86.02 (13)
C17—C18—C13—C12	178.35 (12)	C4—C1—C11—C12	148.28 (12)
C14—C13—C12—O1	106.00 (15)	C2—C1—C11—C12	31.45 (17)
C18—C13—C12—O1	−71.55 (15)	C24—O3—C23—O2	12.4 (2)
C14—C13—C12—C11	−18.3 (2)	C24—O3—C23—C11	−165.93 (13)
C18—C13—C12—C11	164.14 (12)	C10—C11—C23—O2	−5.6 (2)
C19—C18—C17—C22	−0.3 (2)	C12—C11—C23—O2	120.61 (16)
C13—C18—C17—C22	−178.76 (14)	C1—C11—C23—O2	−117.41 (16)
C19—C18—C17—C16	178.06 (14)	C10—C11—C23—O3	172.73 (11)
C13—C18—C17—C16	−0.4 (2)	C12—C11—C23—O3	−61.09 (14)
C2—N2—C3—C8	−177.79 (16)	C1—C11—C23—O3	60.89 (14)
C25—N2—C3—C8	3.1 (3)	C22—C17—C16—C15	178.16 (16)
C2—N2—C3—C4	0.98 (18)	C18—C17—C16—C15	−0.1 (2)
C25—N2—C3—C4	−178.11 (14)	C18—C13—C14—C15	−0.3 (2)
C27—N1—C1—C4	−49.96 (19)	C12—C13—C14—C15	−177.87 (14)
C9—N1—C1—C4	77.10 (15)	C18—C19—C20—C21	0.3 (3)
C27—N1—C1—C2	63.76 (17)	C3—N2—C2—O4	−177.41 (15)
C9—N1—C1—C2	−169.18 (12)	C25—N2—C2—O4	1.7 (3)
C27—N1—C1—C11	−172.81 (14)	C3—N2—C2—C1	−0.36 (17)
C9—N1—C1—C11	−45.76 (14)	C25—N2—C2—C1	178.75 (14)
C8—C3—C4—C5	0.2 (2)	N1—C1—C2—O4	53.2 (2)
N2—C3—C4—C5	−178.66 (14)	C4—C1—C2—O4	176.71 (16)
C8—C3—C4—C1	177.69 (15)	C11—C1—C2—O4	−60.5 (2)
N2—C3—C4—C1	−1.16 (17)	N1—C1—C2—N2	−123.80 (13)
N1—C1—C4—C5	−64.6 (2)	C4—C1—C2—N2	−0.32 (15)
C2—C1—C4—C5	177.95 (17)	C11—C1—C2—N2	122.47 (13)
C11—C1—C4—C5	52.4 (2)	C27—N1—C9—C10	165.99 (14)
N1—C1—C4—C3	118.35 (14)	C1—N1—C9—C10	38.31 (15)
C2—C1—C4—C3	0.88 (15)	C11—C10—C9—N1	−14.51 (16)
C11—C1—C4—C3	−124.70 (13)	C4—C3—C8—C7	0.0 (3)
C17—C18—C19—C20	−0.2 (2)	N2—C3—C8—C7	178.67 (16)
C13—C18—C19—C20	178.25 (15)	C19—C20—C21—C22	0.0 (3)
C9—C10—C11—C23	−127.72 (13)	C3—C4—C5—C6	−0.3 (2)
C9—C10—C11—C12	108.12 (13)	C1—C4—C5—C6	−177.12 (16)
C9—C10—C11—C1	−12.26 (15)	C20—C21—C22—C17	−0.4 (3)
O1—C12—C11—C23	−177.09 (11)	C16—C17—C22—C21	−177.74 (17)
C13—C12—C11—C23	−55.04 (15)	C18—C17—C22—C21	0.6 (3)
O1—C12—C11—C10	−50.22 (15)	C4—C5—C6—C7	0.2 (3)
C13—C12—C11—C10	71.84 (16)	C2—N2—C25—C26	92.5 (2)
O1—C12—C11—C1	63.80 (14)	C3—N2—C25—C26	−88.5 (2)
C13—C12—C11—C1	−174.14 (11)	C17—C16—C15—C14	0.5 (3)
N1—C1—C11—C23	154.27 (11)	C13—C14—C15—C16	−0.2 (3)
C4—C1—C11—C23	28.58 (15)	C5—C6—C7—C8	0.0 (3)
C2—C1—C11—C23	−88.26 (14)	C3—C8—C7—C6	−0.1 (3)
N1—C1—C11—C10	34.47 (13)		

### Hydrogen-bond geometry (Å, º)

**Table:**

*D*—H···*A*	*D*—H	H···*A*	*D*···*A*	*D*—H···*A*
O1—H1···O4	0.82	2.37	2.9121 (16)	124
O1—H1···N1	0.82	2.39	2.9439 (17)	126
